# Phase-Field Damage Modeling of Electromechanical Fracture in MEMS Piezoelectric Films

**DOI:** 10.3390/ma19081662

**Published:** 2026-04-21

**Authors:** Xuanyi Chen, Yuhan Zhang, Yu Xue, Yangjie Shi, Jiaxing Cheng

**Affiliations:** 1 School of Mechanical Engineering, Yangzhou University, Yangzhou 225009, China; mz120230878@stu.yzu.edu.cn (X.C.); mx120250529@stu.yzu.edu.cn (Y.X.); 008178@yzu.edu.cn (Y.S.); 2 School of Mechanics and Engineering, Liaoning Technical University, Fuxin 123000, China; zhangyuhann2002@163.com; 3 Yangzhou Command AI Technology Co., Ltd., Yangzhou 225127, China

**Keywords:** phase-field damage model, MEMS piezoelectric films, crack, polarization direction

## Abstract

**Highlights:**

- A phase-field damage model is developed for crack propagation simulation in MEMS piezoelectric thin films under coupled electromechanical loading.
- The effects of electric potential, polarization angle, and pre-existing defects on the fracture behavior of MEMS devices are quantitatively investigated.
- The proposed model is numerically validated via ABAQUS UEL subroutines for predicting crack initiation and propagation in MEMS structures.
- The model enables accurate simulation of fracture evolution during the fabrication and service of MEMS piezoelectric thin-film devices.

**Abstract:**

Piezoelectric thin films have been widely used in micro-electromechanical systems (MEMSs), such as sensors, actuators, and resonant devices. Electromechanically driven fractures can severely degrade device performance and reliability. In this work, a phase-field damage model is developed for MEMS piezoelectric thin films under coupled electromechanical loading, incorporating pre-existing defects via an equivalent local fracture toughness. Microcracks and micro-voids arising from manufacturing defects are integrated into the model through an effective local fracture toughness, enabling a unified description of their roles in crack initiation and propagation. The proposed model is implemented in ABAQUS by means of a user-defined element (UEL) subroutine and solved using a staggered scheme. Numerical results show that the level of pre-existing defects, the applied electric potential, and the polarization direction all exert significant effects on fracture behavior. As the defect parameter *D_c_* increases from 0 to 0.10, the reaction force decreases from 87.8 N to 86.3 N, indicating reduced fracture resistance due to manufacturing-induced defects. In addition, the reaction force changes from 90.3 N at −500 V to 86.3 N at +500 V, while it decreases from 102.9 N to 87.1 N as the polarization angle *β* increases from 0° to 90°. These results demonstrate that pre-existing defects and electromechanical loading jointly govern crack evolution in MEMS piezoelectric thin films. The present study provides a useful numerical tool for fracture analysis, reliability assessment, and structural design of MEMS piezoelectric devices containing manufacturing defects.

## 1. Introduction

**Figure 1:**
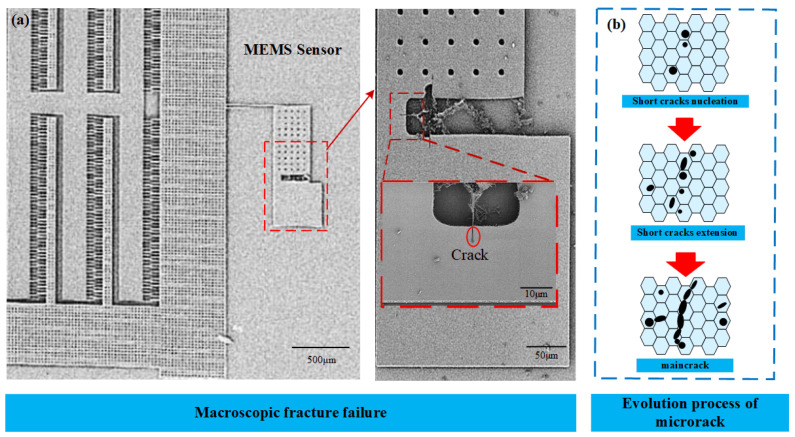
(**a**) Macroscopic fracture failure of MEMS sensor, adapted from Kahn et al. [20] for illustrative purposes; (**b**) evolution process of microcrack.

Micro-electromechanical systems (MEMSs) have been extensively applied across diverse fields, including biomedical devices, automotive systems, communication technologies, and consumer electronics [1,2,3]. Among materials used in MEMS, piezoelectric films are particularly critical owing to their strong electromechanical coupling [4]. These films are commonly used in resonators, accelerometers, pressure sensors, and acoustic devices [5,6,7,8]. During operation, MEMS piezoelectric films are commonly subjected to coupled electromechanical loading. Mechanical loading may arise from external constraints or deformation, while electrical loading is introduced by applied electric potential across the film. Under such combined loading conditions, the interaction between mechanical stress and electric field can strongly affect the initiation and propagation of cracks, thereby influencing the structural integrity and functional performance of the device. MEMS piezoelectric films are often subjected to combined mechanical and electrical loading during operation [9]. Under such conditions, crack initiation and propagation can significantly compromise device performance and reliability [10]. Given the inherent brittleness of piezoelectric materials, understanding fracture behavior is essential for ensuring the safety and reliability of MEMS devices [11]. Therefore, understanding the fracture behavior of MEMS piezoelectric films is essential for reliability assessment and structural design [12]. During the fabrication of MEMS piezoelectric films, defects such as voids, pores, and microcracks may form inside the material or on its surface [13]. These defects can cause local stress concentration, promote crack initiation and propagation under electromechanical loading [14]. As a result, they can compromise the structural integrity and operational reliability of MEMS devices [15]. Although these defects differ in geometry, their effects can be equivalently represented at the continuum scale [16]. Accordingly, they may be treated as local damage sources that reduce fracture resistance [17]. It is necessary to introduce an appropriate equivalent representation of pre-existing defects into the fracture analysis of MEMS piezoelectric films [18]. Figure 1 shows a typical macroscopic fracture failure in a MEMS device and the corresponding evolution of microcracks [19].

Traditional approaches for fracture analysis mainly include classical fracture mechanics and continuum damage mechanics. Classical fracture mechanics, such as Griffith theory and linear elastic fracture mechanics, has been widely used to evaluate crack tip fields, stress intensity factors, and energy release rates for structures containing pre-existing cracks [21,22]. However, these methods usually require the crack path to be known in advance or explicitly tracked during propagation, which makes them less suitable for problems involving spontaneous crack initiation, crack branching, or complex crack trajectories [23]. Continuum damage mechanics, on the other hand, describes material degradation through internal damage variables and is effective in representing distributed stiffness degradation. Nevertheless, it often has difficulty explicitly characterizing discrete crack surfaces and accurately capturing the transition from diffuse material degradation to localized crack propagation. These limitations become more pronounced in coupled multiphysics problems such as piezoelectric fracture, where the interaction between mechanical and electric fields further complicates crack initiation and evolution. Given these challenges, the phase-field method has emerged as an effective and flexible framework for simulating brittle fractures. In recent years, the phase-field method has become a widely used approach for brittle fracture analysis [24,25,26,27]. Existing studies have shown that the method is capable of modeling complex failure mechanisms in composite materials [24], recovering crack opening and sliding responses from the diffused crack description [25], improving computational efficiency for complex crack evolution through adaptive numerical strategies [26], and simulating dynamic crack initiation, propagation, and branching in brittle materials such as advanced ceramics [27]. In addition, robust finite element implementations and variational formulations have further established the phase-field method as an effective framework for brittle fracture simulation [28,29]. Owing to the use of a continuous phase-field variable, crack evolution can be described without explicit crack tracking or repeated remeshing [28,29].

Despite its advantages, the application of the phase-field method to electromechanical fracture presents fundamental numerical and physical challenges that extend beyond purely mechanical cases. A central issue in phase-field modeling is the distinction between crack nucleation and propagation. As highlighted by recent investigations into the variational approach to fracture [30], standard phase-field formulations exhibit a strong sensitivity to the length scale parameter, which can blur the physical boundary between strength-based initiation and toughness-based propagation. This challenge is exacerbated in piezoelectric materials, where the presence of an electric field modifies the driving force for crack evolution. The choice of energy decomposition—specifically how to split electromechanical enthalpy into crack-driving and non-driving—remains an open topic of considerable discussion. Furthermore, the computational cost of resolving diffuse cracks in three-dimensional or complex MEMS geometries demands sophisticated numerical strategies, such as adaptive mesh refinement and robust staggered solution schemes [31], to ensure both accuracy and tractability. Recent comprehensive reviews on multi-physics phase-field fracture [32] have underscored the need for models that can seamlessly handle complex boundary conditions on the crack faces, particularly the transition between permeable and impermeable electrical boundary conditions during crack propagation.

However, most existing studies mainly focus on idealized material systems or defect-free structures, while the role of manufacturing-induced pre-existing defects has received much less attention. This limitation is particularly relevant for MEMS piezoelectric films, where microcracks and voids may significantly affect crack initiation and propagation. To address this gap, the present study introduces an equivalent initial-damage description into the phase-field framework to represent pre-existing defects in MEMS piezoelectric films. This framework has also been extended to piezoelectric materials under electromechanical loading [33,34]. However, most existing studies focus on ideal materials or predefined crack configurations [35,36]. The equivalent representation of pre-existing defects and their effects on fracture evolution remain insufficiently studied in MEMS piezoelectric films [37,38,39]. This limitation restricts the realistic reliability assessment of MEMS devices containing manufacturing imperfections [40]. To address this issue, the present study introduces an equivalent initial-damage description into the phase-field framework to represent pre-existing defects such as microcracks and voids in MEMS piezoelectric films. Based on this formulation, the effects of pre-existing defects on crack initiation and propagation under combined electromechanical loading can be investigated within a unified framework. The proposed model provides a practical numerical approach for fracture analysis and reliability assessment of MEMS devices containing manufacturing imperfections.

The remainder of this paper is organized as follows. Section 2 defines the equivalent initial damage associated with pre-existing defects in piezoelectric materials and presents the formulation of the phase-field model. Section 3 describes the implementation of the proposed model in ABAQUS, with emphasis on the UEL subroutine developed for crack simulation. Section 4 presents numerical examples, including model validation and simulations of fracture behavior under different loading conditions. Section 5 concludes the paper and summarizes the main findings.

## 2. Theoretical Formulation

### 2.1. Equivalent Representation of Pre-Existing Defects

Process variations during fabrication may introduce pre-existing micro-defects, such as micro-voids and microcracks, into MEMS piezoelectric films. These defects are often unavoidable in practice and can significantly compromise the structural integrity and reliability of the devices. To incorporate their effective role into continuum-scale fracture analysis, an RVE-based representation is adopted within the framework of continuum damage mechanics.

**Figure 2:**
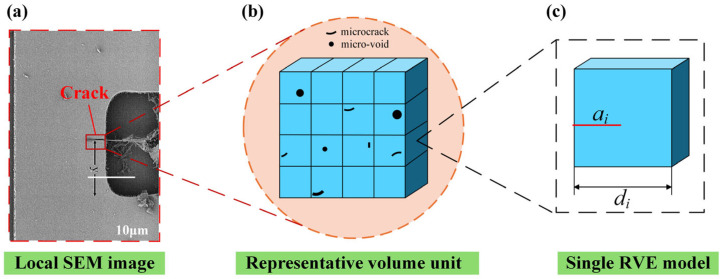
A schematic illustration of pre-existing defects and the representative volume element (RVE): (**a**) a local SEM image of a crack in a MEMS sensor; (**b**) RVE containing pre-existing microcracks and micro-voids; (**c**) equivalent local defect cell characterized by the equivalent crack length *a_i_* and the cell size *d_i_*.

As illustrated in Figure 2, the local SEM image shows a typical crack-related defect in a MEMS sensor, while the RVE schematic is used to represent the distribution of pre-existing microcracks and micro-voids within the material. At the local level, the defect severity is quantified by the equivalent crack length *a_i_* relative to the characteristic element size *d_i_*. Based on this description, an equivalent pre-existing defect parameter is introduced to characterize the local defect level:

(1) $$d_{0}=\frac{S_{D}}{S_{0}}$$

where *S*_0_ denotes the initial area of the RVE, *S_D_* is the equivalent damaged area associated with pre-existing defects.

For a local RVE, the defect level can be expressed as follows:

(2) $$d_{0}^{i}=\frac{a_{i}}{d_{i}}$$

where *a_i_* represents the equivalent crack length associated with the pre-existing defect in the *i*-th local element, and *d_i_* denotes the characteristic size of that element.

Accordingly, the overall defect level of the RVE can be written as follows:

(3) $$d_{0}=\frac{1}{N_{d}}\sum_{i=1}^{N_{d}}d_{0}^{i}$$

where *N_d_* is the number of defect-containing cells within the RVE. Equation (3) gives the overall defect level of the RVE by averaging the local defect contributions of all defect-containing cells. In this way, the effects of micro-voids and microcracks with different local geometries can be represented in a unified manner at the continuum scale.

In the present study, the effect of pre-existing defects is incorporated through an effective local fracture toughness, which is defined as

(4) $$G_{c}^{eff}(x)=G_{c0}(1-d_{0}(x)),$$

where *G_c_*_0_ is the fracture toughness of the defect-free material, and $G_{c}^{eff}(x)$ is the effective local fracture toughness in the presence of pre-existing defects.

It should be noted that the pre-existing defect parameter is not introduced into the initial elastic stiffness tensor. This is because the defects considered in the present work are interpreted as localized manufacturing-induced imperfections that primarily reduce the local fracture resistance, rather than as a homogenized bulk damage field causing significant initial stiffness degradation. Accordingly, their effect is incorporated through the effective local fracture toughness, which more directly reflects their role in facilitating crack initiation and propagation within the phase-field framework.

Equation (4) indicates that pre-existing defects reduce the local fracture resistance of the material. As the defect parameter *d*_0_(x) increases, the effective fracture toughness decreases, which means that crack initiation and propagation become easier in defect-containing regions.

For numerical implementation, the pre-existing defect field is prescribed in regions where manufacturing-induced imperfections are assumed to exist. The defect field is written as follows:

(5) $$d_{c}(x)=\frac{a_{c}}{d_{i}}$$

where *d_c_* is the initial defect level used to quantify the severity of manufacturing-induced defects, and Ω*_d_* denotes the defect region. The parameter *d_c_* is used in the subsequent parametric study to investigate the influence of defect level on fracture behavior.

### 2.2. Electromechanical Constitutive Relations of Piezoelectric Materials

Consider a transversely isotropic piezoelectric material with cracks under two-dimensional conditions in the domain Ω, and let the material boundary be denoted by ∂Ω. The axis of rotational symmetry is assumed to coincide with the local *e*_1_ direction, which is also taken as the polarization direction of the piezoelectric material. Consequently, the material properties on the isotropic plane spanned by *e*_2_ and *e*_3_ are identical.

Under the small-strain assumption, the electromechanical constitutive relations of the piezoelectric material are written as follows:

(6) $$\sigma =C:\varepsilon -e^{T}E$$

(7) D=e:ε+KE

where ***σ*** is the Cauchy stress tensor, ***D*** is the electric displacement vector, **C** is the elastic stiffness tensor, ε is the strain tensor, ***e*** is the third-order piezoelectric tensor in the global coordinate system, ***E*** is the electric field vector and ***K*** is the second-order dielectric tensor.

The strain tensor and the electric field vector ***E*** can be expressed as follows:

(8) $$\varepsilon =\frac{1}{2}(\nabla u+u\nabla )$$

(9) E=−∇ϕ

where ∇ is the Nable operator, ***u*** is the displacement vector and ϕ is the electrical potential.

In the local coordinate system (*e*_1_, *e*_2_), the Cauchy stress tensor $\sigma_{e}^{l}$ and electric displacement $D_{u}^{l}$ can be written as follows:

(10) $$\sigma^{l}=C^{l}:\varepsilon^{l}-e^{lT}E^{l}$$

(11) $$D^{l}=e^{l}:\varepsilon^{l}+K^{l}E^{l}$$

where the superscript *l* denotes quantities defined in the local coordinate system.

The relationship between the elastic stiffness matrix **C** in the global and local coordinate systems **C***^l^* is

(12) $$C=TC^{l}T^{T},$$

where the transformation matrix ***T*** is given by the following:

(13) $$T=\left[\begin{array}{ccc} \cos^{2}\beta & \sin^{2}\beta & -2\cos\beta \sin\beta \\ \sin^{2}\beta & \cos^{2}\beta & 2\cos\beta \sin\beta \\ \cos\beta \sin\beta & -\cos\beta \sin\beta & \cos^{2}\beta -2\sin^{2}\beta \end{array}\right]$$

where β is the rotation angle between the x-axis and the local e1-axis.

Similarly, the third-order piezoelectric tensor **e** and the second-order dielectric tensor ***K*** can be expressed using Voigt notation as follows:

(14) $$e_{ijk}=\alpha_{ii^{\prime }}\alpha_{jj^{\prime }}\alpha_{kk^{\prime }}e_{i^{\prime }j^{\prime }k^{\prime }}^{l}\left(i,j,k=x,y;\;i^{\prime },j^{\prime },k^{\prime }=1,\;2\right)$$

(15) $$K_{ij}=\alpha_{ii^{\prime }}\alpha_{jj^{\prime }}K_{i^{\prime }j^{\prime }}^{l}\left(i,j=x,y;\;i^{\prime },j^{\prime }=1,\;2\right)$$

where the second-order coordinate transformation tensor α can be expressed as

(16) $$\alpha =\left[\begin{array}{cc} \cos\beta & \sin\beta \\ -\sin\beta & \cos\beta \end{array}\right].$$

For the two-dimensional plane strain problem ($\sigma_{33}=0$, $D_{3}=0$) considered in this work, the constitutive relations in Voigt notation are written as follows:

(17) $$\left[\begin{array}{c} \sigma_{11}^{l}\\ \sigma_{22}^{l}\\ \sigma_{12}^{l} \end{array}\right]=\left[\begin{array}{ccc} C_{11} & C_{12} & 0\\ C_{21} & C_{22} & 0\\ 0 & 0 & C_{44} \end{array}\right]\left[\begin{array}{c} \varepsilon_{11}^{l}\\ \varepsilon_{22}^{l}\\ 2\varepsilon_{12}^{l} \end{array}\right]-\left[\begin{array}{cc} e_{11} & 0\\ e_{12} & 0\\ 0 & e_{24} \end{array}\right]\left[\begin{array}{c} E_{1}^{l}\\ E_{2}^{l} \end{array}\right]$$

(18) $$\left[\begin{array}{c} D_{1}^{l}\\ D_{2}^{l} \end{array}\right]=\left[\begin{array}{ccc} e_{11} & e_{12} & 0\\ 0 & 0 & e_{24} \end{array}\right]\left[\begin{array}{c} \varepsilon_{11}^{l}\\ \varepsilon_{22}^{l}\\ 2\varepsilon_{12}^{l} \end{array}\right]+\left[\begin{array}{cc} K_{11} & 0\\ 0 & K_{22} \end{array}\right]\left[\begin{array}{c} E_{1}^{l}\\ E_{2}^{l} \end{array}\right]$$

Unlike conventional damage-based constitutive formulations, the present model does not introduce the pre-existing defect parameter directly into the stress expression. The electromechanical response of the piezoelectric material is described by the standard constitutive relations in Equations (10)–(18), while the influence of pre-existing defects is incorporated through the fracture energy term in the phase-field formulation presented in Section 2.4. This treatment keeps the constitutive structure clear and avoids non-physical stress amplification in highly damaged regions.

### 2.3. Phase-Field Regularization of Brittle Fracture

The variational formulation for sharp cracks has been well addressed by the phase-field model (PFM) which uses a phase-field variable *s* to approximate sharp cracks Γ as diffuse cracks $\Gamma_{s}$. The phase-field variable *s* is equal to 1 in the fully fractured region and 0 in the intact region, while *s* between 0 and 1 represents the transition from the intact region to the fully fractured region.

**Figure 3:**
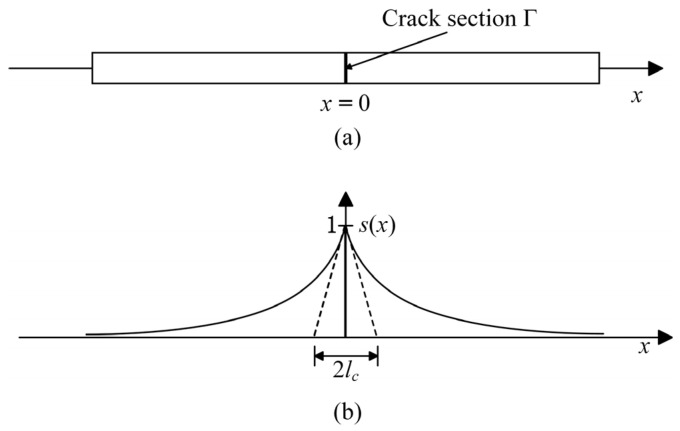
(**a**) One-dimensional sharp crack. (**b**) One-dimensional diffuse crack.

For one-dimensional problems, the sharp and diffuse cracks of the material are shown in Figure 3. The diffuse crack can be approximated as

(19) $$s(x)=\exp^{\left(-\frac{\left|x\right|}{l_{c}}\right)},$$

where *x* = 0 is the position of the crack, and *l*_c_ is the length scale parameter which governs the width of the transition zone between the broken and intact material.

**Figure 4:**
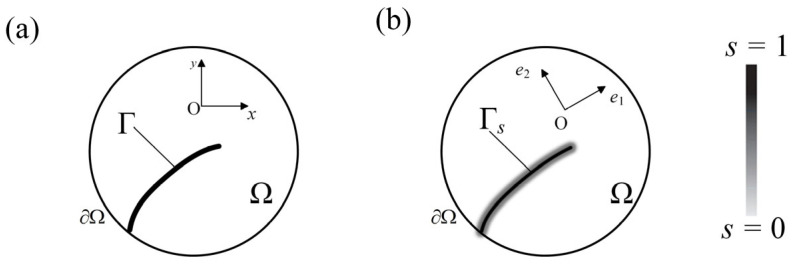
(**a**) Two-dimensional sharp crack. (**b**) Two diffuse cracks.

For two-dimensional problems, the sharp and diffuse cracks of the material are shown in Figure 4. With the help of *s*, sharp crack surface *A* can be approximated to a higher order integral, which can be expressed as follows:

(20) $$A=\int_{\Gamma }dS\approx \int_{\Gamma_{s}}\gamma (s,\nabla s)dV=A_{s}$$

where $A_{s}$ is the diffuse crack surface, and γ(s,∇s) is crack surface density functional which can be defined as follows:

(21) $$\gamma (s,\nabla s)=\frac{1}{2l_{c}}s^{2}+\frac{l_{c}}{2}{\left|\nabla s\right|}^{2}$$

The phase-field variable *s* regularizes the sharp crack topology by introducing a diffusive crack zone of finite width. The length scale parameter *l_c_* controls the width of the transition zone between intact and fractured material. With this regularization, the crack surface can be approximated by a volume integral, which enables the simulation of complex crack initiation and propagation without explicit crack tracking.

To link pre-existing defects with phase-field evolution, the equivalent local crack length is defined as follows:

(22) $$a_{i}=a_{c}+(d_{i}-a_{c})\cdot s_{i}$$

where $a_{i}$ is the equivalent crack length of the *i*-th element, $a_{c}$ is the critical crack length of defect-free material, $d_{i}$ is the initial defect-induced equivalent crack length (Equation (2)), and $s_{i}$ is the phase-field variable. This expression linearly interpolates between the initial defect state ($s_{i}$ = 0, $a_{i}$ = $d_{i}$) and the fully fractured state ($s_{i}$ = 1, $a_{i}$ = $a_{c}$). Substituting ai into Equations (2)–(4) yields a phase-field-dependent effective fracture toughness $G_{c}^{eff}(x,s)$, which is embedded into the subsequent variational phase-field framework to model the coupled effect of manufacturing defects and electromechanical fracture.

### 2.4. Variational Phase-Field Formulation for Electromechanical Fracture

The total potential energy of the cracked body Ω is the sum of the elastic energy, electric potential energy, and fracture energy minus the external work, which can be written as follows:

(23) $$\psi_{\mathrm{total}}=\psi_{\mathrm{bulk}}+\psi_{\mathrm{frac}}-\psi_{\mathrm{ext}}$$

where $\psi_{\mathrm{bulk}}$ is the electromechanical bulk energy, $\psi_{\mathrm{frac}}$ is the fracture energy, and $\psi_{\mathrm{ext}}$ is the external work.

The bulk energy is expressed as follows:

(24) $$\psi_{\mathrm{bulk}}=\int_{\Omega }\varphi_{\mathrm{bulk}}(\varepsilon ,E,s)dV$$

with the bulk energy density defined by the following:

(25) $$\varphi_{\mathrm{bulk}}(\varepsilon ,E,s)=g(s)\varphi_{m}(\varepsilon ,E)+\varphi_{e}(\varepsilon ,E)$$

where $\varphi_{m}$ is the mechanical energy density, and $\varphi_{e}$ denotes the electrical and electromechanical coupling energy contribution.

The degradation function is chosen as

(26) $$g\left(s\right)={\left(1-s\right)}^{2}+k,$$

where *k* is a small positive parameter introduced for numerical stability.

Using the regularized crack surface functional in Equations (15) and (16), the fracture energy can be written as follows:

(27) $$\psi_{\mathrm{frac}}=\int_{\Gamma }G_{c}^{\mathrm{eff}}dS\approx \int_{\Omega }G_{c}^{\mathrm{eff}}\gamma (s,\nabla s)dV=\int_{\Omega }G_{c}^{\mathrm{eff}}\left[\frac{1}{2l_{c}}s^{2}+\frac{l_{c}}{2}{\left|\nabla s\right|}^{2}\right]dV$$

where $G_{c}^{\mathrm{eff}}$ is the effective local fracture toughness defined in Equation (4).

The external work is given by

(28) $$\psi_{\mathrm{ext}}=\int_{\partial \Omega t}h\cdot udA-\int_{\partial \Omega m}M\phi dA,$$

where *h* is the prescribed traction on the mechanical boundary ∂Ωt and *M* is the prescribed traction on the mechanical boundary ∂Ωm.

Substituting Equations (24)–(28) into Equation (23), the total potential energy becomes

(29) $$\begin{array}{c} \psi_{\mathrm{total}}=\int_{\Omega }\left[g\left(s\right)\varphi_{m}+\varphi_{e}+G_{c}^{\mathrm{eff}}\left(\frac{1}{2l_{c}}s^{2}+\frac{l_{c}}{2}{\left|\nabla s\right|}^{2}\right)\right]dV\;\\ -\int_{\partial \Omega t}h\cdot udA+\int_{\partial \Omega m}M\phi dA \end{array}.$$

In the present formulation, the effect of pre-existing defects is introduced through the effective local fracture toughness $G_{c}^{\mathrm{eff}}$, rather than through a direct modification of the constitutive stress relation. This means that manufacturing-induced defects affect fracture evolution by reducing the local resistance to crack growth, while the electromechanical constitutive response remains governed by the standard piezoelectric equations. Such a treatment is physically more consistent for phase-field fracture analysis and avoids the singular stress response that may arise from denominator-type damage corrections.

Within the variational framework, the governing equations are obtained by enforcing the stationarity condition of the total potential energy:

(30) $$\delta \psi_{\mathrm{total}}=0$$

The constitutive relations entering the weak form can then be written as follows:

(31) $$\sigma =g(s)\frac{\partial \varphi_{m}}{\partial \varepsilon }-e^{T}E$$

(32) D=e:ε+KE

Accordingly, the strong forms of the equilibrium equations are as follows:

(33) ∇⋅σ=0, inΩ

(34) ∇⋅D=0, inΩ

The corresponding mechanical and electrical boundary conditions are written as follows:

(35) $$\sigma \cdot n=h,\;\mathrm{on}\;\partial \Omega_{t}$$

(36) $$D\cdot n=-M,\;\mathrm{on}\;\partial \Omega_{m}$$

where **n** is the outward unit normal vector.

To avoid unrealistic crack growth under compression, a tension-compression decomposition of the mechanical energy is adopted. The mechanical energy density is written as

(37) $$\varphi_{m}(\varepsilon ,E)=\varphi_{m}^{+}(\varepsilon ,E)+\varphi_{m}^{-}(\varepsilon ,E),$$

where in the local coordinate system,

(38) $$\varphi_{m}^{+}=\frac{1}{2}\varepsilon_{+}^{l}:C^{l}:\varepsilon_{+}^{l}-\varepsilon_{+}^{l}:e^{lT}\cdot E^{l},$$

(39) $$\varphi_{m}^{-}=\frac{1}{2}\varepsilon_{-}^{l}:C^{l}:\varepsilon_{-}^{l}-\varepsilon_{-}^{l}:e^{lT}\cdot E^{l},$$

where $\varepsilon_{+}^{l}={\left[{\langle \varepsilon_{11}^{l}\rangle }_{+}\;{\langle \varepsilon_{22}^{l}\rangle }_{+}\;2\varepsilon_{12}^{l}\right]}^{T}$ and $\varepsilon_{-}^{l}={\left[{\langle \varepsilon_{11}^{l}\rangle }_{-}\;{\langle \varepsilon_{22}^{l}\rangle }_{-}\;0\right]}^{T}$.

Only the tensile part of the mechanical energy is used to drive phase-field evolution. Therefore, the crack-driving energy density is defined as follows:

(40) $$\psi_{d}=\psi_{m}^{+}$$

To ensure the irreversibility of the evolution process of the phase-field variables, Miehe et al. [28] introduced a history variable field *H* (x, *t*):

(41) $$H(x,t)=\max\varphi_{d}(x,t)$$

Accordingly, the governing equation of the phase-field variable can be written as follows:

(42) $$G_{c}^{\mathrm{eff}}\left[\frac{1}{l_{c}}s-l_{c}\nabla^{2}s\right]-2(1-s)H=0,\;\mathrm{in}\Omega$$

The natural boundary condition for the phase-field variable is

(43) ∇s⋅n=0on ∂Ω.

The governing equations are therefore obtained from the stationarity condition of the total potential energy. To avoid unrealistic crack growth under compression, only the tensile part of the mechanical energy is used to drive phase-field evolution. The irreversibility of crack evolution is enforced through the history field *H* (x, *t*), which stores the maximum crack-driving energy attained during the loading history. As a result, the phase-field evolution equation describes the coupled effects of local fracture resistance, phase-field regularization, and tensile crack-driving energy on crack propagation in defect-containing piezoelectric materials.

For convenience, the crack-driving part of the bulk energy may be rewritten in compact form as

(44) $$\varphi_{d}=\frac{1}{2}\varepsilon_{+}^{l}:C^{l}:\varepsilon_{+}^{l}-\varepsilon_{+}^{l}:e^{lT}\cdot E^{l},$$

and the final phase-field evolution equation is therefore expressed as

(45) $$G_{c}^{\mathrm{eff}}\left[\frac{1}{l_{c}}s-l_{c}\nabla^{2}s\right]-2(1-s)\max\varphi_{d}(x,t)=0.$$

## 3. Numerical Implementation

### 3.1. Finite Element Discretization

Based on the variational framework in Section 2, the coupled electromechanical fracture problem is solved numerically in ABAQUS via a user-defined element (UEL) subroutine. The problem involves three primary unknown fields, namely the displacement field u, the electric potential field ϕ, and the crack phase-field variable s. Their finite element formulation is derived from the weak forms of the mechanical equilibrium equation, the electric balance equation, and the phase-field evolution equation.

The weak form of the mechanical equilibrium equation is written as

(46) $$\int_{\Omega }\sigma :\delta \varepsilon dV=\int_{\partial \Omega_{t}}h\cdot \delta udA,$$

and the weak form of the electric balance equation is expressed as

(47) $$\int_{\Omega }D\cdot \delta EdV=\int_{\partial \Omega_{m}}M\delta \phi dA.$$

The weak form of the phase-field evolution equation is given by

(48) $$\int_{\Omega }G_{c}^{\mathrm{eff}}\left[\frac{1}{l_{c}}s\delta s+l_{c}\nabla s\cdot \delta \nabla s\right]-\int_{\Omega }2(1-s)H\delta sdV=0.$$

Within each element, the phase-field variable s, electric potential ϕ, and displacement u are interpolated as follows:

(49) $$S=\sum_{i=1}^{n}N_{i}^{s}s_{i}=N^{s}s^{e},\;\phi =\sum_{i=1}^{n}N_{i}^{\phi }\phi_{i}=N^{\phi }\phi^{e},\;u=\sum_{i=1}^{n}N_{i}^{u}u_{i}=N^{u}u^{e}$$

and their corresponding gradients are expressed as

(50) $$\nabla s=\sum_{i=1}^{n}B_{i}^{s}s_{i}=B^{s}s^{e},\;\nabla \phi =\sum_{i=1}^{n}B_{i}^{\phi }\phi_{i}=B^{\phi }\phi^{e},\;\nabla u=\sum_{i=1}^{n}B_{i}^{u}u_{i}=B^{u}u^{e},$$

where n is the node index of element; $N_{i}^{s}$, $N_{i}^{\phi }$ and $N_{i}^{u}$ represent the shape functions for the phase-field, electric potential, and displacement fields, respectively. $S_{i}$, $\phi_{i}$, $u_{i}$ represent the nodal values of the phase-field, electric potential, and displacement; $B_{i}^{s}$, $B_{i}^{\phi }$, $B_{i}^{u}$ are the first-order derivative matrices of the shape functions. The matrix forms of the shape functions and their first-order derivatives can be defined as follows:

(51) $$N_{i}^{s}=N_{i}^{\phi }=\left[N_{i}\right],\;N_{i}^{u}=\left[\begin{array}{cc} N_{i} & 0\\ 0 & N_{i} \end{array}\right]$$

(52) $$B_{i}^{s}=B_{i}^{\phi }=\left[\begin{array}{c} N_{i,x}\\ N_{i,y} \end{array}\right],\;B_{i}^{u}=\left[\begin{array}{cc} N_{i,x} & 0\\ 0 & N_{i,x}\\ N_{i,y} & N_{i,x} \end{array}\right]$$

where $N_{i,x}$ and $N_{i,y}$ is the first-order derivatives of the shape function with respect to x and y.

Substituting Equations (49) and (50) into Equations (46)–(47), the residual vectors of the phase-field, electric, and mechanical subproblems are obtained as follows:

(53) $$R^{s}=\int_{\Omega }\left[G_{c}^{\mathrm{eff}}\left(\frac{1}{l_{c}}s{\left(N^{s}\right)}^{T}+l_{c}{\left(B^{s}\right)}^{T}\cdot \nabla s\right)-2(1-s){\left(N^{s}\right)}^{T}H\right]dV$$

(54) $$R^{\phi }=\int_{\Omega }{\left(B^{\phi }\right)}^{T}DdV$$

(55) $$R^{u}=\int_{\Omega }{\left(B^{u}\right)}^{T}\sigma dV$$

The corresponding tangent matrices are expressed as follows:

(56) $$K^{ss}=\int_{\Omega }\left[\frac{G_{c}^{\mathrm{eff}}}{l_{c}}{\left(N^{s}\right)}^{T}N^{s}+G_{c}^{\mathrm{eff}}l_{c}{\left(B^{s}\right)}^{T}B^{s}+2H{\left(N^{s}\right)}^{T}N^{s}\right]dV$$

(57) $$K^{\phi \phi }=-\int_{\Omega }{\left(B^{\phi }\right)}^{T}KB^{\phi }dV$$

(58) $$K^{\phi u}=\int_{\Omega }{\left(B^{\phi }\right)}^{T}eB^{u}dV$$

(59) $$K^{u\phi }=\int_{\Omega }{\left(B^{u}\right)}^{T}e^{T}B^{\phi }dV$$

(60) $$K^{uu}=\int_{\Omega }{\left(B^{u}\right)}^{T}CB^{u}dV$$

### 3.2. Staggered Solution Procedure and UEL Implementation

To solve the strongly coupled electromechanical fracture problem efficiently, a staggered solution strategy is adopted. Within each load increment, the displacement field *u* and electric potential field ϕ are solved simultaneously while the phase-field variable *s* is fixed. The crack-driving history field *H* is then updated from the current electromechanical state, after which the phase-field evolution equation is solved. This procedure is repeated until convergence is achieved for the current increment.

The electromechanical subproblem is expressed in matrix form as follows:

(61) $${\left[\begin{array}{cc} K^{\phi \phi } & K^{\phi u}\\ K^{u\phi } & K^{uu} \end{array}\right]}_{n}{\left[\begin{array}{c} u\\ \phi \end{array}\right]}_{n+1}=-{\left[\begin{array}{c} R^{\phi }\\ R^{u} \end{array}\right]}_{n}$$

and the phase-field subproblem is solved from

(62) $$K_{n}^{ss}S_{n+1}=-R_{n}^{s},$$

where the subscripts n and n + 1 denote the previously converged state and the current iterative state, respectively.

**Figure 5:**
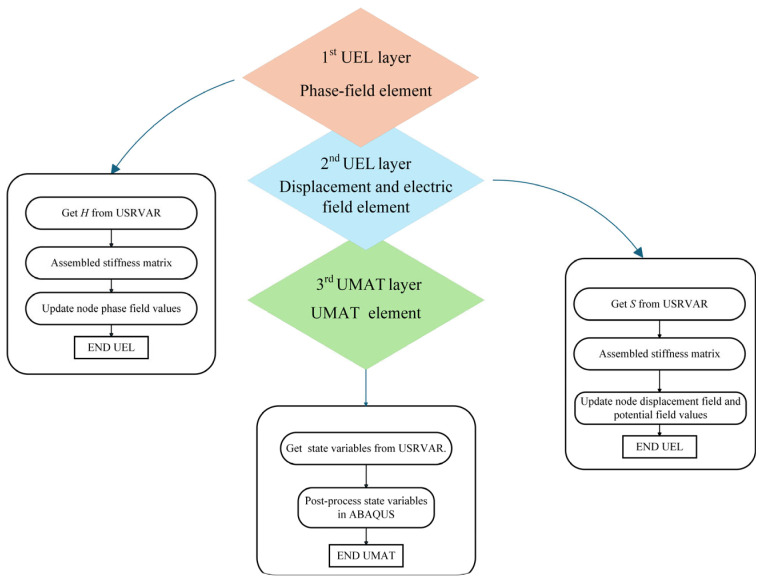
Three-layer finite element structure is implemented in Abaqus using UEL subroutine.

The above staggered algorithm is implemented in ABAQUS using a three-layer finite element structure, as illustrated in Figure 5. The first UEL layer is used to solve the phase-field degree of freedom, the second UEL layer is used to solve the displacement and electric potential degrees of freedom, and the third layer is implemented through UMAT with negligibly small stiffness for field visualization in ABAQUS. The third layer does not contribute to the actual electromechanical response and is introduced solely for post-processing purposes.

During each increment, the electromechanical layer first retrieves the phase-field-related state variables from the shared storage array and assembles the corresponding residual vector and tangent matrix. After updating the displacement and electric potential fields, the crack-driving history field is evaluated and passed to the phase-field layer. The phase-field layer then assembles its own residual vector and tangent matrix and solves the corresponding evolution equation. Information exchange among the three layers is realized through shared nodes and user-defined state variables, ensuring consistent coupling among the displacement, electric potential, and phase-field fields.

To enforce crack irreversibility, the history field is updated according to the following formula:

(63) $$H(x,t_{n+1})=\max(H(x,t_{n}),\varphi_{d}(x,t_{n+1}))$$

In the numerical implementation, a small residual stiffness parameter *k* is retained in the degradation function to maintain numerical stability in the fully damaged region. For each load increment, the staggered iterations are repeated until the prescribed convergence criterion is satisfied. The increment size and the maximum number of iterations are selected to ensure stable crack evolution and robust convergence of the coupled solution.

For clarity, the state variables stored in the user subroutine are summarized in Table 1. These variables mainly include the phase-field value, crack-driving energy history, electric potential, and auxiliary quantities required for inter-layer data transfer.

## 4. Numerical Results and Discussion

### 4.1. Benchmark Validation for a Purely Elastic Single-Edge-Notched Plate

**Figure 6:**
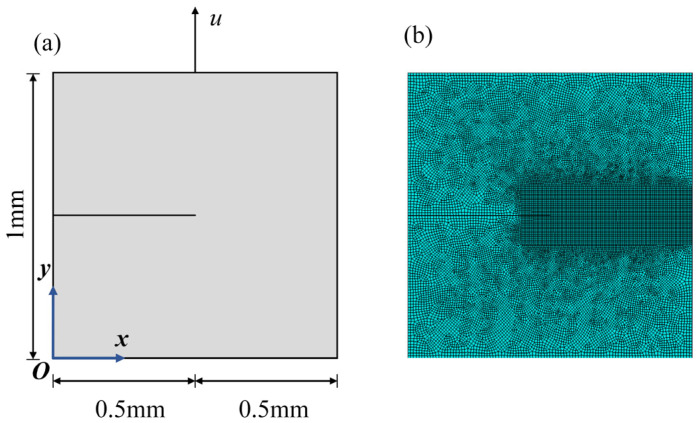
(**a**) Boundary conditions of the purely elastic material; (**b**) finite element mesh of the purely elastic material.

**Figure 7:**
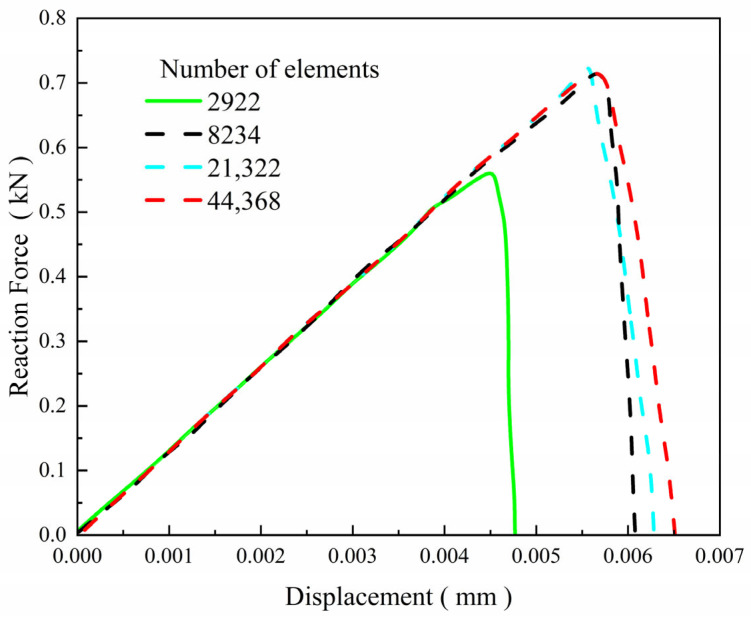
Displacement–load curves for different numbers of elements.

**Figure 8:**
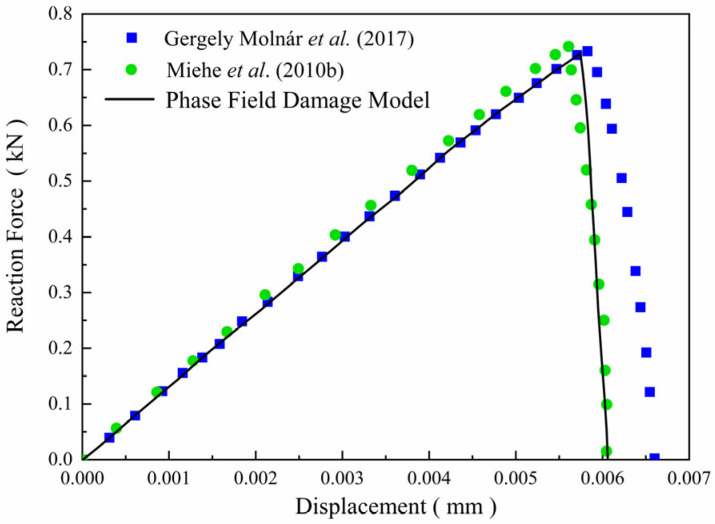
Reaction force–displacement curve of single-edge-notched square plate [28,29].

The proposed phase-field formulation is first validated through comparison with literature results [28] for a single-edge-notched square plate under purely elastic conditions. The geometry and boundary conditions of the model are illustrated in Figure 6. The bottom boundary of the plate is fixed in both the x- and y-directions, while a monotonically increasing vertical displacement is prescribed on the top boundary. The displacement loading is imposed incrementally with a step size of Δu = 1 × 10^−7^ mm. The material properties are taken as an elastic modulus *E* = 210 GPa and a Poisson’s ratio *ν* = 0.3, and the phase-field length scale parameter is set to *l_c_* = 0.0075 mm. The reaction force–displacement response obtained from the present implementation is shown in Figure 7 and compared with the reference results reported by Miehe et al. [28]. In addition, a mesh sensitivity analysis was performed using four different meshes containing 2922, 8234, 21,322, and 44,368 elements, respectively. As shown in Figure 7, the numerical results gradually converge with mesh refinement. In particular, the responses obtained with 8234, 21,322, and 44,368 elements are in very close agreement, indicating that the mesh with 8234 elements is already sufficient to provide accurate results. Therefore, considering both computational accuracy and efficiency, the mesh with 8234 elements was adopted in the subsequent simulations. As shown in Figure 8, the reaction force–displacement curves obtained from the present model and the reference results show close agreement during the loading process. In particular, the peak reaction force predicted by the present model is 729 N, whereas the corresponding values reported in the literature are 741 N and 733 N, giving relative differences of only 1.62% and 0.55%, respectively. These quantitative comparisons demonstrate that the present finite element implementation can accurately reproduce the benchmark fracture response predicted by the classical phase-field formulation, thereby confirming the reliability of the numerical implementation used in this study.

### 4.2. Electromechanical Fracture of a Piezoelectric Single-Edge-Notched Plate

**Figure 9:**
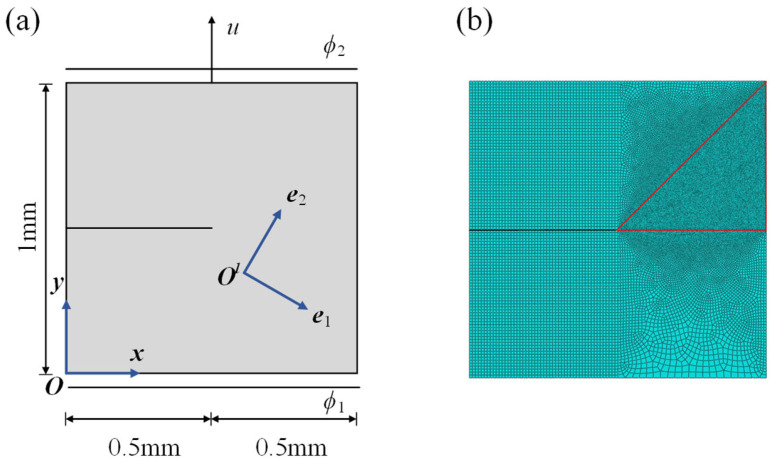
(**a**) Boundary conditions of single-edge-notched piezoelectric square plate; (**b**) Finite element mesh of single-edge-notched piezoelectric square plate (The red-selected area is the grid encryption area).

After verifying the correctness of the numerical implementation for purely elastic materials, the proposed model is applied to simulate the fracture behavior of a piezoelectric single-edge-notched square plate subjected to coupled electromechanical loading. The geometry, boundary conditions, and finite element mesh of the model are shown in Figure 9. A prescribed vertical displacement is applied at the top boundary, while the electric potentials at the bottom and top boundaries are specified as *ϕ*_1_ and *ϕ*_2_, respectively. The local material coordinate system is rotated by *β* = 45° relative to the global coordinate system. The numerical model is discretized using 33,709 finite elements. The piezoelectric material considered in the simulation is PZT-4, and the corresponding material parameters are listed in Table 2.

**Figure 10:**
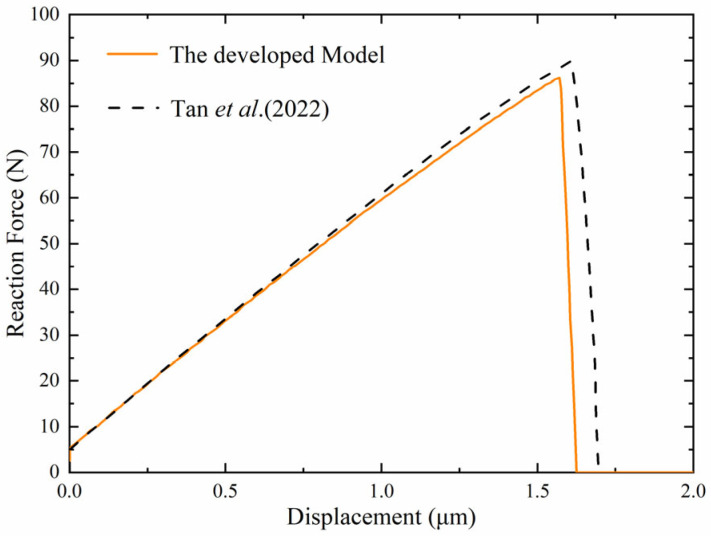
Reaction force–displacement curve of single-edge-notched piezoelectric square plate [41].

The reaction force–displacement response of the notched plate is presented in Figure 10, where the present results are compared with reference results reported in the literature. In addition to the purely elastic benchmark, this comparison also serves to validate the proposed formulation for piezoelectric fracture under coupled electromechanical loading. As shown in Figure 10, the present model predicts an earlier reduction in the load-carrying capacity of the structure. This difference is mainly attributed to the introduction of equivalent initial defects through the effective local fracture toughness, which reduces the fracture resistance near the notch tip and allows the crack-driving energy to reach the critical level at a smaller displacement. As a result, crack initiation occurs earlier and the global response enters the softening stage sooner than in the reference solution. Although slight differences are observed near the peak load, the overall trend of the numerical response remains consistent with the published results, confirming that the proposed model can reasonably capture the electromechanical fracture behavior of piezoelectric materials containing manufacturing-induced imperfections.

### 4.3. Effect of Applied Electric Potential on Crack Propagation

**Figure 11:**
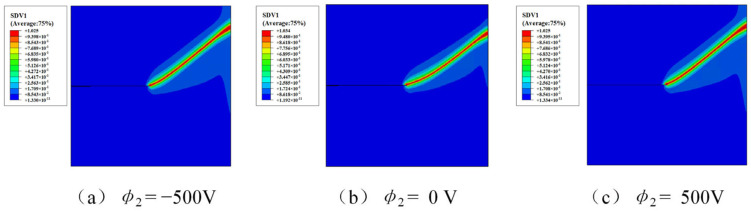
The crack paths for various applied electrical potentials with *β* = 45° and *D*_c_ = 0.1. (**a**) *ϕ*_2_ = −500 V; (**b**) *ϕ*_2_ = 0 V; (**c**) *ϕ*_2_ = 500 V. (SDV1 represents the phase field value).

**Figure 12:**
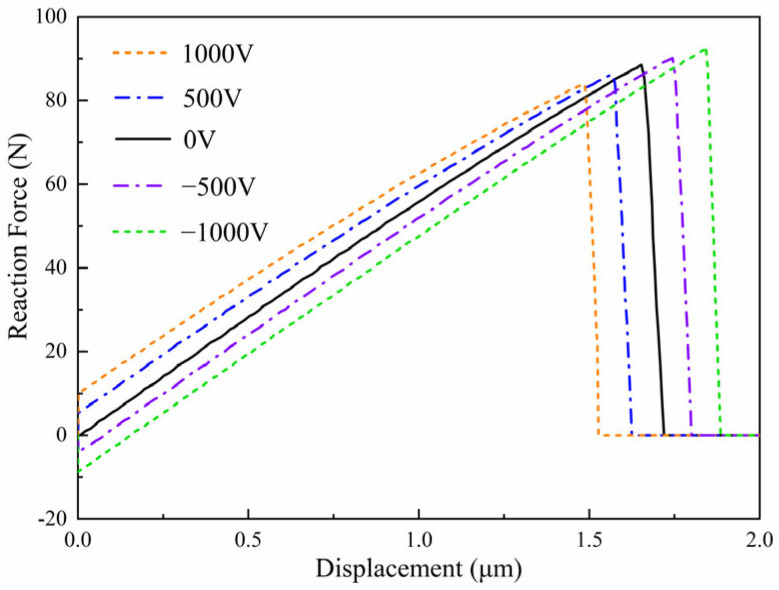
Reaction force–displacement curve for different electric potentials with *ϕ*_2_ = ± 500 V and *ϕ*_2_ = 0 V.

**Figure 13:**
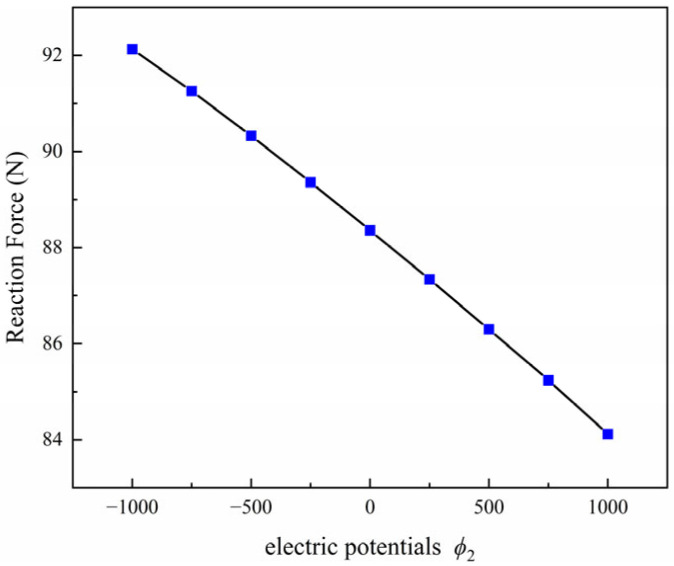
Variation in reaction force for different electric potentials with *β* = 45°and *D_c_* = 0.1.

The influence of electrical loading on crack propagation is investigated by applying different electric potentials at the top boundary of the plate. Three electrical boundary conditions are considered in this study, namely *ϕ*_2_ = −500 V, *ϕ*_2_ = 0 V, and *ϕ*_2_ = 500 V, while the electric potential at the bottom boundary is fixed at *ϕ*_1_ = 0 V. The polarization direction is taken as *β* = 45°, and the equivalent initial defect parameter is set as *D_c_* = 0.1. The predicted crack paths under different electric potentials are presented in Figure 11, and the corresponding reaction force–displacement curves are shown in Figure 12. The variation in reaction force for different electric potentials is shown in Figure 13.

The results shown in Figure 11, Figure 12 and Figure 13 indicate that the applied electric potential influences both the crack trajectory and the electromechanical response of the structure. In the present parameter range, the differences between the force–displacement curves in Figure 11 remain relatively small, while slight variations in crack propagation behavior can still be observed from the crack paths in Figure 10 and the reaction force variation in Figure 12. This behavior can be explained by the fact that mechanical displacement loading is the dominant driving factor for crack propagation, while the electric potential mainly modifies the local electromechanical coupling near the crack tip. Different electrical boundary conditions alter the distribution of the electric field and consequently affect the crack-driving energy density. Therefore, although the overall trend of the global mechanical response remains similar, the applied electric potential still produces observable differences in crack initiation and propagation behavior.

### 4.4. Effect of Polarization Direction on Crack Propagation

**Figure 14:**
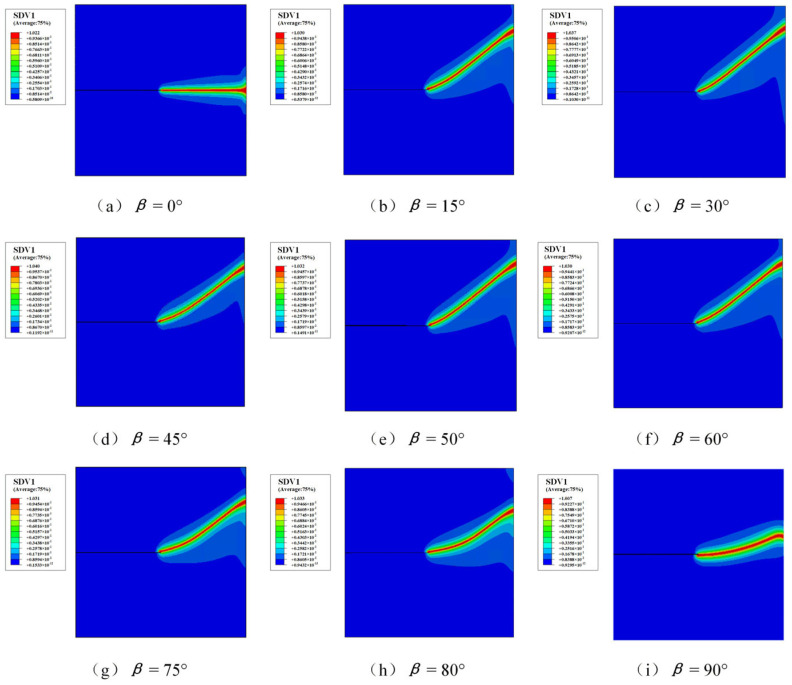
The crack paths for various rotation angles *β* with *ϕ*_2_ = 500 V and *D_c_* = 0.1. (**a**) *β* = 0°; (**b**) *β* = 15°; (**c**) *β* = 30°; (**d**) *β* = 45°; (**e**) *β* = 50°; (**f**) *β* = 60°; (**g**) *β* = 75°; (**h**) *β* = 80°; (**i**) *β* = 90°. (SDV1 represents the phase field value).

The polarization direction of piezoelectric materials plays an important role in determining the fracture behavior under coupled electromechanical loading. To investigate this effect, different polarization angles *β* are considered while keeping the electrical boundary condition fixed at *ϕ*_2_ = 500 V and the equivalent initial defect parameter at *D_c_* = 0.1. The predicted crack paths corresponding to different polarization angles are shown in Figure 14.

**Figure 15:**
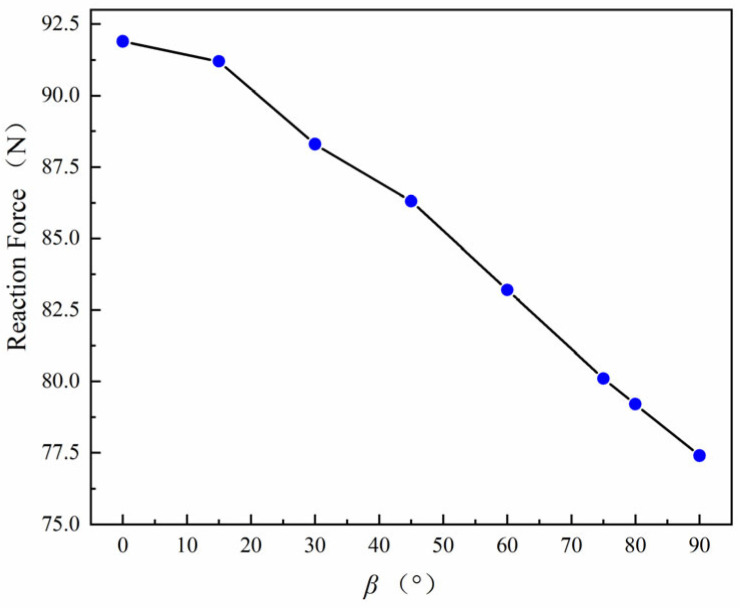
Variation in reaction force with polarization angle *β* with *ϕ*_2_ = 500 V and *D_c_* = 0.1.

It can be observed that the crack propagation trajectory changes with the polarization direction. This phenomenon is associated with the electromechanical coupling behavior of the piezoelectric material. The orientation of the direction of polarization modifies the coupling terms in the constitutive relations, which in turn affects the distribution of electromechanical energy around the crack tip. The variation in the reaction force with respect to the polarization angle is shown in Figure 15. The results indicate that the reaction force gradually decreases as the polarization angle increases within the considered range. This implies that the load required for crack propagation becomes smaller when the polarization direction deviates from the loading direction, leading to a reduction in the fracture resistance of the structure.

### 4.5. Effect of Equivalent Initial Damage on Crack Propagation

**Figure 16:**
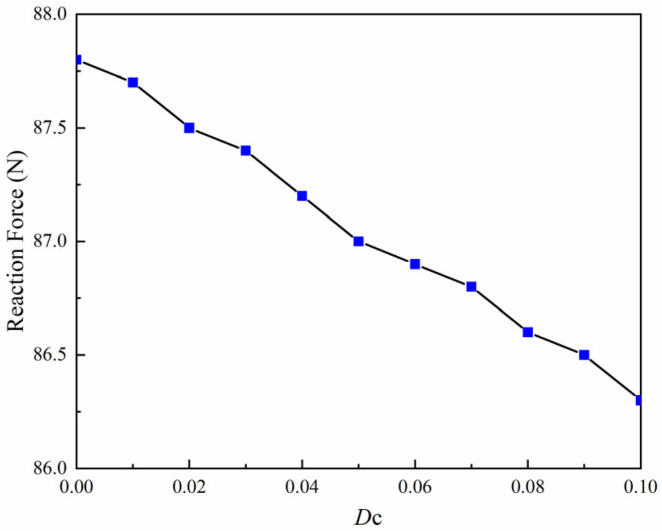
Variation in reaction force with equivalent initial defect parameter *D_c_* under *ϕ*_2_ = 500 V and *β* = 45°.

The effect of equivalent initial defects on crack propagation is further investigated by varying the defect parameter Dc while keeping the electrical boundary condition and polarization direction fixed. In this analysis, the electric potential is set to *ϕ*_2_ = 500 V, and the polarization direction is taken as *β* = 45°. The variation in reaction force with the equivalent defect parameter *D_c_* is shown in Figure 16.

The results show that the reaction force decreases monotonically as the defect parameter *D_c_* increases. In the present formulation, a larger *D_c_* corresponds to a lower effective local fracture toughness, which reduces the resistance of the material to crack growth. Consequently, crack initiation occurs at a smaller displacement, and the propagation process becomes easier in regions containing higher levels of pre-existing defects. From a physical perspective, manufacturing-induced imperfections such as micro-voids, pores, and microcracks can act as local weakening zones in MEMS piezoelectric films. Although these defects are not explicitly modeled as sharp cracks, their collective influence is represented through the equivalent defect parameter introduced in the present formulation. The numerical results therefore demonstrate that the defect level plays an important role in determining the fracture behavior of MEMS piezoelectric devices.

## 5. Conclusions

In this study, a phase-field fracture model incorporating an equivalent representation of pre-existing defects was developed for MEMS piezoelectric thin films under coupled electromechanical loading and implemented in ABAQUS through a UEL subroutine. The main conclusions can be summarized as follows.

(1) The proposed model establishes a reliable numerical framework for simulating crack initiation and propagation in MEMS piezoelectric films containing fabrication-induced defects. By introducing pre-existing defects through an equivalent local reduction in fracture toughness, the model can account for manufacturing-related damage in a unified and physically consistent manner.
(2) The equivalent initial defect level has a pronounced influence on fracture behavior. As the defect parameter *D_c_* increases from 0 to 0.10, the reaction force decreases from 87.8 N to 86.3 N, indicating that higher defect levels reduce fracture resistance and facilitate crack initiation and propagation.
(3) The fracture response is also strongly affected by electromechanical coupling conditions. The reaction force decreases from 102.9 N to 87.1 N as the polarization angle *β* increases from 0° to 90°, while it changes from 90.3 N at −500 V to 86.3 N at +500 V under different applied electric potentials. These results demonstrate that defect level, electric loading, and polarization orientation jointly govern crack evolution in MEMS piezoelectric thin films.

## Figures and Tables

**Table 1:** List of state variables.

Variable	SDVs
Phase-field element	
Phase-field variables *s*	SDV1
Strain energy *ψ* + *m*	SDV2
Electromechanical element	
Phase-field variables *s*	SDV3
Strain energy *ψ* + *m*	SDV4
Electrical potential *ϕ*	SDV5

**Table 2:** Material properties for PZT-4.

Property		Value	
Elastic constants (GPa)		*c*_11_ = 115 *c*_12_ = 74.3	*c*_21_ =74.3
		*c*_44_ = 25.6	
Piezoelectric (Cm^−2^)		*e*_11_ = 13.84 *e*_12_ = −6.98	*e*_24_ = 13.44
Dielectric (10^−9^ Fm^−1^)		*K*_11_ = 5.47 *K*_22_ = 6	
Length scale parameter (mm)	*l_c_* = 0.01		

## Data Availability

The original contributions presented in this study are included in the article. Further inquiries can be directed to the corresponding author.
